# Di(2-ethylhexyl) Phthalate Metabolites May Alter Thyroid Hormone Levels in Men

**DOI:** 10.1289/ehp.9852

**Published:** 2007-03-12

**Authors:** John D. Meeker, Antonia M. Calafat, Russ Hauser

**Affiliations:** 1 Department of Environmental Health Sciences, University of Michigan, Ann Arbor, Michigan, USA; 2 National Center for Environmental Health, Centers for Disease Control and Prevention, Atlanta, Georgia, USA; 3 Department of Environmental Health, Harvard School of Public Health, Boston, Massachusetts, USA; 4 Vincent Memorial Obstetrics and Gynecology Service, Andrology Laboratory and In Vitro Fertilization Unit, Massachusetts General Hospital, Boston, Massachusetts, USA

**Keywords:** biomarkers, endocrine disruption, epidemiology, hormone, phthalates, thyroid, urinary metabolites

## Abstract

**Background:**

Phthalates are used extensively in many personal-care and consumer products, resulting in widespread nonoccupational human exposure through multiple routes and media. A limited number of animal studies suggest that exposure to phthalates may be associated with altered thyroid function, but human data are lacking.

**Methods:**

Concurrent samples of urine and blood were collected from 408 men. We measured urinary concentrations of mono(2-ethylhexyl) phthalate (MEHP), the hydrolytic metabolite of di(2-ethylhexyl) phthalate (DEHP), and other phthalate monoester metabolites, along with serum levels of free thyroxine (T_4_), total triiodothyronine (T_3_), and thyroid-stimulating hormone (TSH). Oxidative metabolites of DEHP were measured in urine from only 208 of the men.

**Results:**

We found an inverse association between MEHP urinary concentrations and free T_4_ and T_3_ serum levels, although the relationships did not appear to be linear when MEHP concentrations were categorized by quintiles. There was evidence of a plateau at the fourth quintile, which was associated with a 0.11 ng/dL decrease in free T_4_ [95% confidence interval (CI), –0.18 to –0.03] and a 0.05 ng/mL decrease in T_3_ (95% CI, –0.10 to 0.01) compared with the first (lowest) MEHP quintile. The inverse relationship between MEHP and free T_4_ remained when we adjusted for oxidative metabolite concentrations; this simultaneously demonstrated a suggestive positive association with free T_4_.

**Conclusions:**

Urinary MEHP concentrations may be associated with altered free T_4_ and/or total T_3_ levels in adult men, but additional study is needed to confirm the observed findings. Future studies must also consider oxidative DEHP metabolites relative to MEHP as a potential marker of metabolic susceptibility to DEHP exposure.

Human exposure to some industrial compounds may result in adverse health outcomes mediated through the neuroendocrine axis. These chemicals may affect the synthesis, secretion, transport, binding, action, or elimination of natural hormones in the human body that are responsible for maintaining homeostasis, reproduction, development, and/or behavior [U.S. Environmental Protection Agency (EPA) 1997]. In addition to being essential for normal brain development, thyroid hormones play an important role in many physiologic systems, and alterations in thyroid hormone levels can lead to a myriad of adverse clinical conditions (Nussey and Whitehead 2001). Although much is still unknown about mechanisms and consequences involved with the relationship between environmental exposures and changes in thyroid hormone levels, phthalates and other environmental chemicals may bind to thyroid receptors and influence thyroid hormone signaling (Zoeller 2005).

Phthalates are used extensively in many personal-care and consumer products, resulting in widespread nonoccupational human exposure through multiple routes and media (Hauser and Calafat 2005). High-molecular-weight phthalates [e.g., di(2-ethylhexyl) phthalate (DEHP)], are primarily used as plasticizers in the manufacture of flexible vinyl, which is then used in consumer products, flooring and wall coverings, food contact applications, and medical devices [Agency for Toxic Substances and Disease Registry (ATSDR) 2002; Hauser and Calafat 2005]. Low-molecular-weight phthalates [e.g., diethyl phthalate (DEP), dibutyl phthalate (DBP)] are used in personal-care products (e.g., perfumes, lotions, cosmetics), as solvents and plasticizers for cellulose acetate, and in formulating lacquers, varnishes, and coatings, including those used to provide timed releases in some pharmaceuticals (ATSDR 2001; Hauser and Calafat 2005). The Centers for Disease Control and Prevention’s (CDC) *Third National Report on Human Exposure to Environmental Chemicals* (CDC 2005) showed that the majority of males in the United States have detectable concentrations of several phthalate monoesters in urine [monoethyl phthalate (MEP), mono(2-ethyl-hexyl) phthalate (MEHP), monobutyl phthalate (MBP), and monobenzyl phthalate (MBzP)], reflecting widespread exposure to the parent diester compounds among the general population. Two oxidative metabolites of DEHP, mono-(2-ethyl-5-hydroxylhexyl) phthalate (MEHHP) and mono-(2-ethyl-5-oxohexyl) phthalate (MEOHP), were present in most subjects at urinary concentrations higher than those of MEHP, the hydrolytic metabolite of DEHP (CDC 2005).

Animal studies have shown that some phthalates, namely DBP, butylbenzyl phthalate (BBzP), and DEHP, cause testicular toxicity and other adverse male reproductive health outcomes (ATSDR 2001, 2002; Hauser and Calafat 2005), whereas human studies on phthalate exposure and male reproductive health have been inconsistent (Duty et al. 2003a, 2003b; Hauser et al. 2006; Hauser and Calafat 2005; Jonsson et al. 2005; Murature et al. 1987; Rozatti et al. 2002). Studies investigating the association between exposure to phthalates and thyroid function are limited. In animal studies, rats with diets contaminated with DEHP were found to have thyroid alterations and lower plasma thyroxine (T_4_) concentrations compared with controls (Hinton et al. 1986; Howarth et al. 2001; Poon et al. 1997; Price et al. 1988). In addition, a recent *in vitro* study reported that DEHP and other phthalates caused changes in the iodide uptake of thyroid follicular cells (Wenzel et al. 2005). A dose-dependent inverse association between DBP and both triiodothyronine (T_3_) and T_4_ has also been reported in male rats (O’Connor et al. 2002). We are unaware of human studies on phthalates and thyroid function; therefore, we designed the present study to investigate potential relations between biological markers of phthalate exposure and levels of T_4_, T_3_, and thyroid-stimulating hormone (thyrotrophin, TSH) in adult men.

## Methods

Subjects were recruited from an ongoing study on the relationship between environmental agents and male reproductive health. They were men who were partners in subfertile couples seeking evaluation between January 2000 and May 2004 from the Vincent Burnham Fertility Center at Massachusetts General Hospital (Boston, MA). The study was approved by the Human Studies Institutional Review Boards of the Massachusetts General Hospital, Harvard School of Public Health, the CDC, and the University of Michigan. After the study procedures were explained and all questions answered, subjects signed an informed consent. Men 18–55 years of age, who were not postvasectomy, were eligible to participate. Of those approached, approximately 65% consented. Most men who declined to participate in the study cited lack of time on the day of their clinic visit as the reason for not participating.

### Phthalate metabolites in urine

On the day of each subject’s clinic visit, a single spot urine sample was collected into a sterile specimen cup prescreened for phthalates. Phthalate metabolites were measured in urine because of potential sample contamination from the parent diester and because the metabolites, as opposed to the parent diesters, are believed to be the active toxicants (Li et al. 1998; Peck and Albro 1982). The analytical approach for the analysis of the urinary phthalate monoester metabolites (i.e., MEHP, MBP, MBzP, and MEP) and two oxidative metabolites of DEHP (i.e., MEHHP and MEOHP) involved enzymatic deconjugation of the metabolites from their glucuronidated form, solid-phase extraction, separation with HPLC, and detection by isotope-dilution tandem mass spectrometry (Blount et al. 2000; Silva et al. 2003, 2004b). Detection limits were in the low nanogram per milliliter range and varied slightly by analytical batch for each phthalate metabolite (MEP, 1.00–1.21 ng/mL; MBP, 0.60–1.07 ng/mL; MBzP, 0.47–1.0 ng/mL; MEHP, 0.87–1.20 ng/mL; MEHHP, 0.95–1.60 ng/mL; MEOHP, 1.07–1.20 ng/mL). We used isotopically labeled internal standards and conjugated internal standards to increase precision of measurements. Along with the unknown samples, each analytical run included calibration standards, reagent blanks, and quality control materials of high and low concentrations to monitor for accuracy and precision. Analysts at the CDC were blind to all information concerning subjects. Urinary phthalate metabolite concentrations were adjusted for urine dilution by specific gravity (SG) using the following formula:


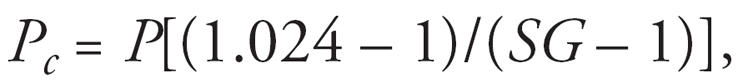


where *P_c_* is the *SG*-adjusted phthalate metabolite concentration (nanograms per milliliter), *P* is the observed phthalate metabolite concentration, and *SG* is the specific gravity of the urine sample. *SG* was measured using a hand-held refractometer (National Instrument Company, Inc., Baltimore, MD).

Using the urinary concentrations of the three DEHP metabolites (i.e., MEHP, MEHHP, and MEOHP), we calculated the percentage of these DEHP metabolites excreted as the hydrolytic monoester (MEHP%). We consider the MEHP% a phenotypic marker of the proportion of DEHP metabolized to and excreted in the urine as MEHP. The greater the MEHP%, the larger the percentage of DEHP excreted as MEHP relative to the excretion of the two oxidative metabolites. To calculate MEHP%, we converted MEHP, MEHHP, and MEOHP concentrations to nanomoles per milliliter; divided MEHP concentrations by the sum of concentrations of MEOHP, MEHHP, and MEHP; and multiplied by 100. To our knowledge, the use of MEHP% as a phenotypic marker of DEHP metabolism and excretion is novel and has not been used in human health studies until recently (Hauser et al. 2006).

### Thyroid hormones and TSH

One non-fasting blood sample was drawn between 0900 and 1600 hours on the same day the urine sample was collected. Blood samples were centrifuged and serum stored at –80°C until analysis. Free T_4_, total T_3_, and TSH concentrations were determined in serum by microparticle enzyme immunoassay using an automated immunoassay system (AxSYM; Abbott Diagnostics, Abbott Park, IL). The assay sensitivities for free T_4_ and total T_3_ were 0.01 ng/dL and 0.15 ng/mL, respectively. The interassay coefficients of variation (CVs) for both hormones were < 9%. For TSH, the ultrasensitive hTSH II assay was used with a functional sensitivity of 0.03 μIU/L and interassay CVs of < 8%.

### Statistical analysis

Data analysis was performed using SAS version 9.1 (SAS Institute Inc., Cary, NC). Descriptive statistics on subject demographics were tabulated, along with the distributions of phthalate metabolite concentrations and thyroid hormones. For phthalate metabolite values below the limit of detection (LOD), an imputed value equal to one-half the LOD was used. Thyroid hormone and phthalate metabolite concentrations were stratified by demographic categories to investigate the potential for confounding. We used multivariate linear regression to explore relationships between thyroid hormones and urinary phthalate metabolite concentrations. Concentrations of T_4_ and T_3_ closely approximated normality and were used in statistical models untransformed; the distribution of TSH concentration was skewed left and was therefore log-transformed for statistical analyses. SG-adjusted phthtalate metabolite concentrations were also log-transformed. Inclusion of covariates was based on statistical and biologic considerations (Kleinbaum et al. 1998). Age and body mass index (BMI) were modeled as a continuous variable; smoking status was dichotomized by current smoker versus never smoked or former smoker; and race was categorized into four groups: white, African American, Hispanic, and other. We considered previous examination for infertility (yes or no), prior impregnation of a partner (yes or no), and timing of blood/urine samples by season (winter vs. spring, summer, or fall) and time of day (0900–1259 hours vs. 1300–1600 hours) for inclusion in the models as dichotomous variables. To improve interpretability, the regression coefficients were back transformed and expressed as a change in the dependent variable (i.e., hormone levels) for an interquartile range (IQR) increase in phthalate metabolite concentrations. For MEHP, we also included MEHHP or MEHP% in the models, in addition to an interaction term, to explore evidence of whether individual differences in DEHP metabolism alter susceptibility to MEHP. Because, as expected, MEHHP and MEOHP are highly correlated (*r* = 0.98), the results for models including MEHHP were identical to those with MEOHP and are therefore not presented. Our hypothesis is that the concentrations of MEHHP (or MEOHP) and/or MEHP% may represent phenotypic markers for efficient or inefficient metabolism of DEHP to its oxidative metabolites. In secondary analyses, the multivariate models were rerun after excluding men with highly concentrated or highly dilute urine samples (SG > 1.03 or < 1.01) (Teass et al. 1998). Finally, we also assessed nonlinear relationships between phthalate metabolite concentrations and hormones by regressing the hormones on quintiles of phthalate metabolites.

## Results

Of the 478 men with phthalate metabolites measured in urine, 422 had free T_4_, total T_3_, and TSH levels measured in serum. An additional 14 subjects taking hormone medications (e.g., propecia, finasteride, cabergoline, clomid, gonadotropin-releasing hormone, testosterone, prednisone taper) were excluded from the present analysis. In addition, none of the men reported taking medications that may alter the thyroid axis (i.e., amidoarone, carbamazepine, chlorpropamide, carbidopa/levodopa, heparin, interferon, lithium, phenytoin, phenobarbital, propylthiouracil, sulfasalazine, synthroid). Among the remaining 408 subjects (Table 1), most were white (85%) and had never smoked (72%). The mean (± SD) age and BMI were 36 ± 5.3 years and 28 ± 4.5, respectively. Distributions of SG-adjusted phthalate metabolite concentrations are presented in Table 2, and distributions of the thyroid hormones and TSH measured in serum are presented in Table 3. Among the 408 urine samples, MEP, a metabolite of diethyl phthalate, was detected in 100% of the samples, whereas MBP and MBzP were detected in > 97% and > 94% of the samples, respectively. Of the samples, 83% had detectable concentrations of MEHP. The sample size for MEOHP and MEHHP was 208 because analytical methods for the quantification of these analytes were only recently implemented in this study. More than 95% of these samples had detectable concentrations of MEHHP and MEOHP. Spearman correlations between MEHP and MEHHP or MEOHP were 0.74 and 0.71, respectively.

Age was inversely associated with both free T_4_ and total T_3_ (Spearman correlation coefficients were –0.2 and –0.1, respectively; *p* < 0.05 for both), whereas there were suggestive positive weak associations of BMI with T_3_, TSH, MBzP, and both oxidative DEHP metabolites (all Spearman correlation coefficients were 0.1; all *p*-values were between 0.05 and 0.1). Current smokers had higher median T_3_ levels (1.04 ng/mL) and lower median TSH (1.1 μIU/mL) than never-smokers (0.96 ng/mL and 1.5 μIU/mL, respectively). Smoking status was not associated with T_4_. Samples collected in winter had median T_4_ concentrations slightly lower than those collected in spring, summer, or fall (1.1 vs. 1.2 ng/dL), and median TSH was higher in samples collected in the morning compared with samples collected in the afternoon (1.5 vs. 1.4 μIU/mL). For the SG-adjusted phthalate metabolites, current smokers had higher median concentrations of MEP (215 ng/mL) but lower concentrations of MEHHP and MEOHP (21 ng/mL and 16 ng/mL, respectively) compared with never-smokers (140, 45, and 32 ng/mL respectively). As previously observed (Silva et al. 2004a), median MEHP concentrations were also higher among men whose urine samples were collected in the afternoon (9.4 ng/mL) compared with men who provided urine samples in the morning (6.9 ng/mL).

Crude regression results were similar to the adjusted results, with the exception of MEP, where there was a suggestive inverse association with total T_3_ (*p* = 0.07) and a suggestive positive association with TSH (*p* = 0.1) that were no longer evident when covariates were included in the models (*p* = 0.2). Results from the multivariate regression analyses are presented in Table 4. All models were adjusted for age, BMI, smoking, and the time of day blood/urine samples were collected. We found an inverse association between SG-adjusted urinary MEHP concentration and serum total T_3_ levels, where an IQR increase in MEHP was associated with a 0.021-ng/mL decrease in T_3_ [95% confidence interval (CI), –0.042 to –0.001 ng/mL; *p* = 0.04]. For the median level of T_3_ (0.96 ng/mL), this represents a 2.2% decrease in T_3_ for an IQR increase in MEHP (3.16–21.3 ng/mL). In sensitivity analyses, effect estimates from the multivariate models were similar when men with SG outside the acceptable range were excluded (*n* = 339; results not shown).

When the analysis was limited to the subset of men with oxidative DEHP metabolite measures (*n* = 208), the inverse association between MEHP and T_3_ became weaker. An IQR increase in MEHP was associated with a 0.011-ng/mL decrease in T_3_ (95% CI, –0.021 to 0.009 ng/mL; *p* = 0.4). However, there was an inverse association between MEHP% and free T_4_ (Table 4). An IQR increase in MEHP% was associated with a 0.030-ng/dL decrease in free T_4_ (95% CI –0.055 to –0.005 ng/dL; *p* = 0.02). For the median T_4_ level (1.2 ng/dL), this represents a 2.5% decrease in T_4_ for an IQR increase in MEHP% (6% to 17% MEHP). When both MEHP% and MEHP were included in the models, the inverse association between MEHP% and T_4_ remained (Table 5). When MEHHP and MEHP were both included in the models, T_4_ was inversely associated with MEHP but positively associated with MEHHP. There was no evidence of collinearity between MEHP and MEHHP (i.e., the SEs and 95% CIs were not inflated). Results were identical when MEOHP was included in the models instead of MEHHP (data not shown), because MEHHP and MEOHP concentrations were highly correlated. Results for T_3_ followed the same pattern as free T_4_ when both MEHP and MEHHP were included in the model, although associations were weaker and not statistically significant (Table 5). We explored the interaction terms (MEHP × MEHHP or MEHP × MEHP%), but we found no evidence of interaction when these terms were added to the multivariate models.

**Figure 1:**
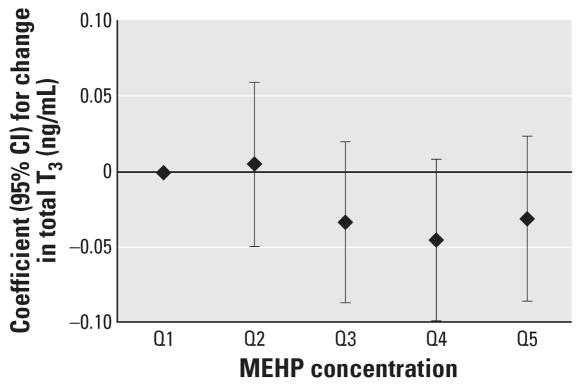
Regression coefficients (95% CIs) for a change in total T_3_ associated with increasing quintiles of SG-adjusted MEHP. Adjusted for age, BMI, smoking, and time of day (*n* = 408).

**Figure 2:**
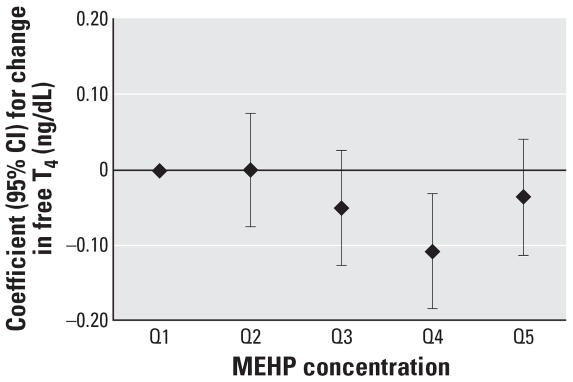
Regression coefficients (95% CIs) for a change in free T_4_ associated with increasing quintiles of SG-adjusted MEHP. Adjusted for age, BMI, smoking, and time of day (*n* = 408).

**Figure 3:**
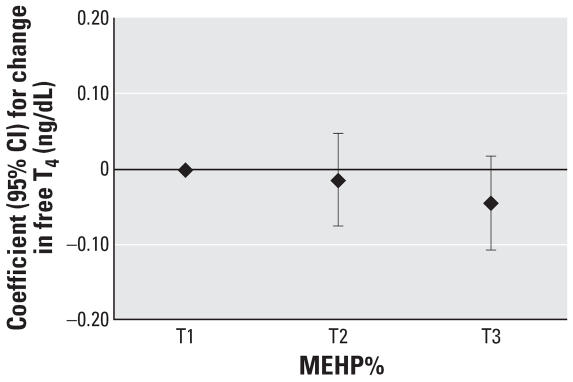
Regression coefficients (95% CIs) for a change in free T_4_ associated with increasing tertiles of MEHP%. Adjusted for age, BMI, smoking, and time of day (*n* = 208).

**Figure 4:**
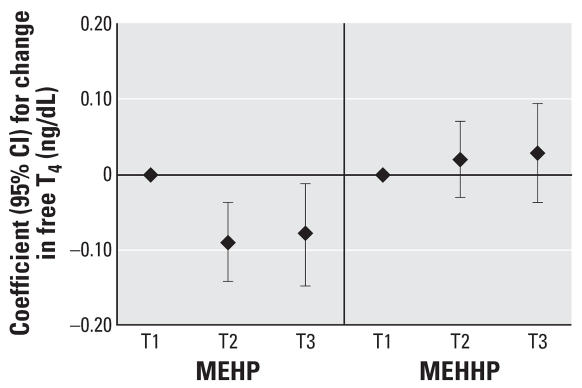
Regression coefficients (95% CIs) for a change in free T_4_ associated with increasing tertiles of MEHP and MEHHP. Adjusted for age, BMI, smoking, and time of day (*n* = 208).

**Figure 5:**
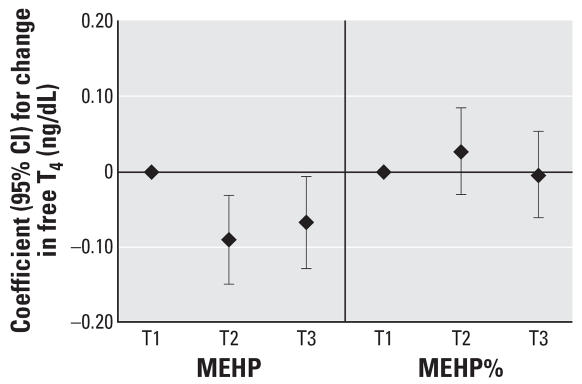
Regression coefficients (95% CIs) for a change in free T_4_ associated with increasing tertiles of MEHP and MEHP%. Adjusted for age, BMI, smoking, and time of day (*n* = 208).

To assess the robustness of the associations and potential nonlinear relationships, we categorized SG-adjusted phthalate concentrations into quintiles (*n* = 408). We found a suggestive inverse trend for MEHP quintiles and T_3_ (*p* = 0.07), whereas we unexpectedly found a significant inverse trend for MEHP quintiles and free T_4_ (*p* = 0.04). The regression coefficients for increasing quintiles of MEHP appeared to plateau at quintile 4 (Figures 1 and 2). Among the subset of men with MEHP oxidative metabolite measures (*n* = 208), metabolite concentrations were categorized by tertiles because of the smaller sample size. The tertile analysis resulted in a suggestive inverse trend between MEHP% and free T_4_ (*p* = 0.07; Figure 3). When tertiles of both MEHP and MEHHP were included, there was an inverse association between free T_4_ and medium and high tertiles of MEHP compared with the lowest MEHP tertile (Figure 4). Similar to the quintile analysis among all the men, the relationships did not appear to be linear but were suggestive of having a plateau. Results were similar when MEHP and MEHP% were included in the same model (Figure 5). In addition, the suggestive association between MEHP% and free T_4_ was no longer evident.

We compared concentrations of (unadjusted) urinary phthalate metabolites measured in the present study with those among U.S. males published in the *Third National Report on Human Exposure to Environmental Chemicals* (CDC 2005). Metabolite distributions were generally similar between the present study and the national data, although we found slightly lower concentrations of MEP, MBP, and MBzP. Conversely, urinary concentrations of MEHP, MEHHP, and MEOHP were somewhat higher in the present study than those from the Third National Report. For example, the median and 95th percentile values for MEHP (unadjusted for SG) in the present study were 6.3 and 112 ng/mL, respectively, compared with 4.3 and 37.9 ng/mL in the Third National Report (CDC 2005).

## Discussion

To our knowledge, this is the first study to investigate the association between environmental exposures to phthalates and serum thyroid hormone and TSH levels in humans. In the present study, we found evidence for an inverse association between urinary MEHP concentrations comparable with those reported for the general U.S. population, and free T_4_ and total T_3_ levels in adult men (CDC 2005). When metabolite concentrations were modeled as continuous variables, there was an inverse association between MEHP and T_3_. We also found evidence of a plateau in the inverse association for both T_3_ and free T_4_ with MEHP when exposure was categorized by quintiles, suggesting that the relationship is nonlinear; regression approaches that assume linearity may not be appropriate for assessing the relationship between MEHP and thyroid hormones. Nonmonotonic inverse associations with a plateau or even a U shape may be plausible, as has been demonstrated at low doses for other hormonally active compounds (Welshons et al. 2003).

The associations between MEHP and thyroid hormones in the present study are somewhat consistent with limited animal studies, in which rats fed DEHP-contaminated diets had histopathologic thyroid changes consistent with hyperactivity and decreased T_4_ concentrations compared with controls, whereas T_3_ levels remained essentially unchanged (Hinton et al. 1986; Howarth et al. 2001; Poon et al. 1997; Price et al. 1988). Conversely, rats intravenously administered DEHP, at concentrations representing the amount that can leach from polyvinyl chloride blood bags used for human blood transfusions, showed increased serum T_3_ and T_4_ levels (Gayathri et al. 2004). This may suggest differences in DEHP toxicokinetics by route of exposure (ingestion vs. intravenous), although evidence for this has not been shown in other studies to date. In another study in rats fed a DEHP-contaminated diet (2%) for 21 days, Bernal et al. (2002) reported no difference in T_4_ levels between exposed and control groups. However, serum T_4_ levels were lower in exposed rats (3.44 ± 0.53 ng/mL) than in controls (4.20 ± 0.58 ng/mL); the lack of statistical significance may have been due to a small number of animals in each group (*n* = 12 and 7, respectively).

Virtually every tissue in the body is affected by the thyroid hormones (Vander et al. 1998), but studies on adverse health effects associated with small deficits of T_4_ or T_3_ in humans are currently lacking (Boelart and Franklyn 2005; Surks et al. 2004). There are a number of potential mechanisms by which environmental chemicals can affect thyroid function and disrupt thyroid hormone homeostasis—including involvement of the sodium–iodide symporter, thyroid peroxidase enzyme, receptors for thyroid hormones or TSH, and transport proteins or cellular uptake mechanisms—all of which may interfere with the hypothalamic–pituitary–thyroid axis at different levels (Boas et al. 2006). Interaction between environmental chemicals and iodothyronine deiodinases or hepatic enzymes may also influence peripheral metabolism of thyroid hormones (Qatanani et al. 2005). Limited evidence exists that phthalates may be involved in a number of these mechanisms. Recent *in vitro* studies suggest that phthalates induce changes in iodide uptake of thyroid follicular cells by altering the transcriptional activity of the sodium–iodide sym-porter (Breous et al. 2005; Wenzel et al. 2005). Ishihara et al. (2003) found that, in birds, phthalates bind competitively to transthyretin, a major thyroid hormone–binding transport protein. In addition, Shimada and Yamauchi (2004) found that phthalates (DBP and BBzP, but not DEHP) inhibited [^125^I]T_3_ uptake in tadpole red blood cells. Additional research is needed for a better understanding of how phthalates may influence the endocrine system and thyroid function. Alternatively, at this time we cannot rule out the possibility of reverse causation whereby thyroid status may affect DEHP and/or MEHP metabolism in a manner that results in the observed inverse associations between DEHP metabolites and thyroid hormones. A limitation of the present study was that we did not measure levels of total T_4_ and free T_3_; these measurements, in conjunction with free T_4_ and total T_3_ levels, may have provided additional mechanistic insights.

MEHP%, which provides information on the proportion of DEHP excreted in the urine as the monoester compared with the oxidative metabolites but does not provide information on the magnitude of DEHP exposure, was inversely associated with free T_4_ (Table 4). In addition, when both MEHP and oxidative metabolites were considered in multivariate models, we found an inverse association between MEHP and free T_4_ along with evidence of a positive association between MEHHP and free T_4_ (Table 5, Figure 4). These results suggest that the efficiency of an individual’s ability to oxidize DEHP to MEHHP and MEOHP may be related to increased circulating free T_4_ levels. For instance, for a given urinary concentration of MEHP, an increased urinary concentration of MEHHP or MEOHP was associated with increased circulating free T_4_. Conversely, for a given urinary concentration of MEHHP or MEOHP, an increased MEHP concentration is associated with decreased free T_4_. This phenomenon was also reflected in the inverse association between MEHP% and free T_4_, where having an increased percentage of total DEHP urinary metabolites excreted as MEHP was associated with decreased T_4_ levels. This suggests that for a given concentration of MEHP, the higher the percentage of DEHP excreted as MEHP (i.e., the more MEHP there is relative to MEHHP and MEOHP in the urine), the lower the serum T_4_. These results may indicate that the pathways by which DEHP is metabolized may impart variable risks for altered thyroid hormones, based on the proportion of DEHP that undergoes oxidative metabolism compared with the proportion that is excreted as the monoester.

An understanding of the metabolism of DEHP may provide insights into our observations on the relationships between DEHP metabolites and thyroid hormone levels. DEHP hydrolyzes first to MEHP, which subsequently metabolizes to MEHHP and MEOHP, among other oxidative metabolites (Koch et al. 2005; Silva et al. 2006a, 2006b). These DEHP oxidative metabolites are more easily excreted in urine than MEHP. Therefore, oxidation of MEHP could effectively decrease internal body burden of MEHP, which in turn, may have a protective effect if MEHP is the bioactive metabolite. DEHP interindividual variability in the percentage of MEHP and of oxidative metabolites that are excreted in the urine has been observed in the present study (Table 2) and elsewhere (Becker et al. 2004; CDC 2005; Hauser et al. 2006; Silva et al. 2006a). Therefore, because the proportion of urinary excretion of DEHP as MEHP varies across individuals, urinary concentrations of MEHP alone do not represent total body burden of DEHP exposure. The inclusion of oxidative metabolites such as MEHHP and MEOHP, as shown by our results for free T_4_, may provide additional insights into DEHP exposure and metabolism.

An alternative explanation is that the relative percentage of DEHP oxidative metabolites in urine may represent a surrogate for the function of phase 1 enzymes. If other hormonally active chemicals requiring phase 1 enzymes for detoxification are associated with thyroid function or thyroid hormone levels, men with high MEHP%, which represents low functionality of the phase 1 enzymes, may also be “poor” metabolizers of the other hormonally active chemicals. Presently, there is no evidence to support this, although it remains a possible alternative explanation.

To our knowledge, other studies have not explored the associations of thyroid hormone levels with urinary phthalate monoester metabolites or with oxidative metabolites of DEHP. Further investigation is warranted on the association between MEHP and thyroid hormones, and the potential utility of MEHP% as a phenotypic marker of the proportion of DEHP excreted as MEHP and its oxidative metabolites. As with other phthalates, interindividual variability in DEHP metabolism and urinary excretion of metabolites exists (Becker et al. 2004; CDC 2005; Hauser et al. 2006; Silva et al. 2006a). Furthermore, the timing of collection of the urine sample may partially account for differences in urinary concentrations of MEHP and the oxidative metabolites among individuals because the oxidative metabolites have a longer half-life than MEHP (Koch et al. 2005). For instance, a urine sample collected a few hours after DEHP exposure would contain primarily MEHP. By contrast, a urine sample collected 12 hr after DEHP exposure may have higher concentrations of MEHHP and MEOHP than of MEHP. The differences in half-lives of DEHP metabolites should be taken into account when interpreting the meaning of MEHP% following a single pulsed exposure to DEHP. However, the interpretation of MEHP% would be more straightforward if the differences in half-lives were not as influential on urinary concentrations, as in the case of chronic exposure to DEHP.

## Conclusion

In the present study we found that urinary MEHP concentrations comparable with those found among the general U.S. population (CDC 2005) may be associated with altered free T_4_ and/or T_3_ levels in adult men. However, this is the first report of these associations in humans; thus, additional research is necessary to substantiate the observed findings. Future studies must also consider oxidative DEHP metabolites relative to MEHP as a potential marker of metabolic susceptibility to DEHP exposure.

## Figures and Tables

**Table 1:** Subject demographics (*n* = 408).

Characteristic	Mean ± SD	No. (%)
Age (years)	36.2 ± 5.34	
BMI^a^	27.8 ± 4.52	
Race		
White		346 (85)
Black/African American		15 (4)
Hispanic		19 (5)
Other		28 (7)
Smoking^b^		
Never-smoker		292 (72)
Ever-smoker		113 (28)
Current smoker		38 (9)
Former smoker		75 (18)
Season of blood sample		
Winter		111 (27)
Spring, summer, fall		297 (73)
Time of blood sample		
0900–1259 hours		171 (42)
1300–1600 hours		237 (58)

a Information on BMI missing for 2 subjects.

b Information on smoking status missing for 3 subjects.

**Table 2:** Distribution of SG-adjusted phthalate metabolites in urine (ng/mL).

			Percentiles						
Phthalate metabolite	No.	GM	10th	25th	50th	75th	90th	95th	Max
MEP	408	184	29.8	59.9	158	535	1,391	2,343	11,371
MBP	408	16.7	5.01	10.4	17.0	30.4	45.6	65.1	14,459
MBzP	408	7.70	2.40	4.20	8.16	15.7	24.9	42.4	520
MEHP	408	8.28	0.96	3.16	7.95	21.3	66.7	127	876
MEHHP	208	58.2	13.4	23.4	48.9	113	332	786	4,805
MEOHP	208	38.5	8.80	16.3	32.9	71.3	228	497	3,063
MEHP%	208	9	3	6	10	17	25	31	61

Abbreviations: GM, geometric mean; max, maximum.

**Table 3:** Distribution of thyroid hormones and TSH in serum (*n* = 408).

		Percentiles						
Hormone	Mean^a^	5th	10th	25th	50th	75th	90th	95th
Free T_4_ (ng/dL)	1.25	0.94	0.99	1.08	1.20	1.40	1.56	1.72
Total T_3_ (ng/mL)	0.97	0.70	0.76	0.83	0.96	1.08	1.21	1.25
TSH (μIU/mL)	1.43	0.62	0.77	1.04	1.43	1.95	2.77	3.58

a Arithmetic mean presented for free T_4_ and total T_3_, and geometric mean presented for TSH.

**Table 4:** Adjusted^a^ regression coefficients (95% CI) for thyroid hormones associated with an interquartile range (IQR) increase in SG-adjusted urinary phthalate metabolite concentrations (*n* = 408).

Phthalate metabolite^b^	Free T_4_^c^	Total T_3_^c^	TSH^d^,^e^
MEP	–0.011 (–0.048 to 0.026)	0.018 (–0.009 to 0.044)	0.94 (0.85 to 1.03)
MBP	0.003 (–0.023 to 0.028)	–0.005 (–0.024 to 0.012)	1.02 (0.96 to 1.09)
MBzP	–0.017 (–0.046 to 0.011)	0.001 (–0.018 to 0.021)	1.01 (0.94 to 1.08)
MEHP	–0.013 (–0.042 to 0.017)	–0.021 (–0.042 to –0.001)*	0.97 (0.90 to 1.04)
MEHHP^f^	0.008 (–0.017 to 0.033)	–0.002 (–0.030 to 0.025)	0.98 (0.88 to 1.08)
MEOHP^f^	0.013 (–0.010 to 0.035)	0.003 (–0.024 to 0.028)	0.97 (0.88 to 1.06)
MEHP%^f^	–0.030 (–0.055 to –0.005)*	–0.016 (–0.044 to 0.014)	1.04 (0.94 to 1.15)

a Adjusted for age, BMI, current smoking, and time of day blood sample was collected.

b Natural log-transformations of urinary concentrations of phthalate metabolites were used in all models.

c Coefficient represents the change in hormone level for an IQR change in phthalate metabolite concentration after back-transformation of the phthalate metabolite concentrations; for an IQR change in phthalate metabolite concentration, a coefficient equal to 0 indicates no change in hormone level, a coefficient < 0 indicates a decrease in hormone level, and a coefficient > 0 indicates an increase in hormone level.

d Log-transformations of TSH concentration was used; free T_4_ and total T_3_ concentrations were modeled untransformed.

e Coefficient represents a multiplicative change in hormone level for an IQR change in phthalate metabolite concentration after back-transformation of both hormone and phthalate metabolite concentrations; for an IQR change in phthalate metabolite concentration, a coefficient equal to 1.0 indicates no change in hormone level, a coefficient < 1.0 indicates a multiplicative decrease in hormone level, and a coefficient > 1.0 indicates a multiplicative increase in hormone level.

f *n* = 208.

* *p* < 0.05.

**Table 5:** Adjusted^a^ regression coefficients for a change in thyroid hormones associated with an IQR increase in MEHP when also adjusted for MEHHP or MEHP% (*n* = 208).

	Free T_4_		Total T_3_		TSH^b^	
	Estimate (95%CI)	*p*-Value	Estimate (95%CI)	*p*-Value	Estimate (95%CI)	*p*-Value
MEHP^b^	–0.046 (–0.088 to –0.004)	0.03	–0.027 (–0.074 to 0.021)	0.27	1.05 (0.89 to 1.25)	0.57
MEHHP^b^,^c^	0.043 (0.002 to 0.083)	0.04	0.017 (–0.028 to 0.065)	0.44	0.94 (0.80 to 1.11)	0.48
MEHP^b^	0.008 (–0.021 to 0.038)	0.59	–0.004 (–0.040 to 0.031)	0.80	0.97 (0.86 to 1.10)	0.66
MEHP%^b^,^d^	–0.034 (–0.065 to –0.004)	0.02	–0.012 (–0.047 to 0.022)	0.46	1.05 (0.93 to 1.19)	0.41

a Adjusted for age, BMI, current smoking, and time of day blood sample was collected.

b Variable was natural log-transformed in the models.

c Coefficient represents change in thyroid hormone associated with IQR increase in MEHHP adjusted for MEHP.

d Coefficient represents change in thyroid hormone associated with IQR increase in MEHP% adjusted for MEHP.

## References

[b1-ehp0115-001029] ATSDR (2001). Toxicological Profile for Di-n-butyl Phthalate (DBP).

[b2-ehp0115-001029] ATSDR (2002). Toxicological Profile for Di(2-ethylhexyl)phthalate (DEHP).

[b3-ehp0115-001029] Becker K, Seiwert M, Angerer J, Heger W, Koch HM, Nagorka R (2004). DEHP metabolites in urine of children and DEHP in house dust. Int J Hyg Environ Health.

[b4-ehp0115-001029] Bernal CA, Martinelli MI, Mocchiutti NO (2002). Effect of the dietary exposure of rat to di(2-ethyl hexyl) phthalate on their metabolic efficiency. Food Addit Contam.

[b5-ehp0115-001029] Blount BC, Milgram KE, Silva MJ, Malek NA, Reidy JA, Needham LL (2000). Quantitative detection of eight phthalate metabolites in human urine using HPLC-APCI-MS/MS. Anal Chem.

[b6-ehp0115-001029] Boas M, Feldt-Rasmussen U, Skakkebaek NE, Main KM (2006). Environmental chemicals and thyroid function. Eur J Endocrinol.

[b7-ehp0115-001029] Boelaert K, Franklyn JA (2005). Thyroid hormone in health and disease. J Endocrinol.

[b8-ehp0115-001029] Breous E, Wenzel A, Loos U (2005). The promoter of the human sodium/iodide symporter responds to certain phthalate plasticisers. Mol Cell Endocrinol.

[b9-ehp0115-001029] CDC (2005). Third National Report on Human Exposure to Environmental Chemicals.

[b10-ehp0115-001029] Duty SM, Silva MJ, Barr DB, Brock JW, Ryan L, Chen Z (2003a). Phthalate exposure and human semen parameters. Epidemiology.

[b11-ehp0115-001029] Duty SM, Singh NP, Silva MJ, Barr DB, Brock JW, Ryan L (2003b). The relationship between environmental exposures to phthalates and DNA damage in human sperm using the neutral comet assay. Environ Health Perspect.

[b12-ehp0115-001029] Gayathri NS, Dhanya CR, Indu AR, Kurup PA (2004). Changes in some hormones by low doses of di (2-ethyl hexyl) phthalate (DEHP), a commonly used plasticizer in PVC blood storage bags & medical tubing. Indian J Med Res.

[b13-ehp0115-001029] Hauser R, Calafat AM (2005). Phthalates and human health. Occup Environ Med.

[b14-ehp0115-001029] Hauser R, Meeker JD, Duty SM, Silva MJ, Calafat AM (2006). Altered semen quality in relation to urinary concentrations of phthalate monoester and oxidative metabolites. Epidemiology.

[b15-ehp0115-001029] Hinton RH, Mitchell FE, Mann A, Chescoe D, Price SC, Nunn A (1986). Effects of phthalic acid esters on the liver and thyroid. Environ Health Perspect.

[b16-ehp0115-001029] Howarth JA, Price SC, Dobrota M, Kentish PA, Hinton RH (2001). Effects on male rats of di-(2-ethylhexyl) phthalate and di-n-hexylphthalate administered alone or in combination. Toxicol Lett.

[b17-ehp0115-001029] Ishihara A, Nishiyama N, Sugiyama S, Yamauchi K (2003). The effect of endocrine disrupting chemicals on thyroid hormone binding to Japanese quail transthyretin and thyroid hormone receptor. Gen Comp Endocrinol.

[b18-ehp0115-001029] Jonsson BA, Richthoff J, Rylander L, Giwercman A, Hagmar L (2005). Urinary phthalate metabolites and biomarkers of reproductive function in young men. Epidemiology.

[b19-ehp0115-001029] Kleinbaum DG, Kupper LL, Muller KE, Nizam A (1998). Selecting the best regression equation. Applied Regression Analysis and Other Multivariate Methods.

[b20-ehp0115-001029] Koch HM, Bolt HM, Preuss R, Angerer J (2005). New metabolites of di(2-ethylhexyl)phthalate (DEHP) in human urine and serum after single oral doses of deuterium-labelled DEHP. Arch Toxicol.

[b21-ehp0115-001029] Li LH, Jester WF, Orth JM (1998). Effects of relatively low levels of mono-(2-ethylhexyl) phthalate on cocultured Sertoli cells and gonocytes from neonatal rats. Toxicol Appl Pharmacol.

[b22-ehp0115-001029] Murature DA, Tang SY, Steinhardt G, Dougherty RC (1987). Phthalate esters and semen quality parameters. Biomed Environ Mass Spectrom.

[b23-ehp0115-001029] Nussey S, Whitehead S (2001). Endocrinology: An Integrated Approach.

[b24-ehp0115-001029] O’Connor JC, Frame SR, Ladics GS (2002). Evaluation of a 15-day screening assay using intact male rats for identifying anti-androgens. Toxicol Sci.

[b25-ehp0115-001029] Peck CC, Albro PW (1982). Toxic potential of the plasticizer di(2-ethylhexyl) phthalate in the context of its disposition and metabolism in primates and man. Environ Health Perspect.

[b26-ehp0115-001029] Poon R, Lecavalier P, Mueller R, Valli VE, Procter BG, Chu I (1997). Subchronic oral toxicity of di-n-octyl phthalate and di(2-ethyl-hexyl) phthalate in the rat. Food Chem Toxicol.

[b27-ehp0115-001029] Price SC, Chescoe D, Grasso P, Wright M, Hinton RH (1988). Alterations in the thyroids of rats treated for long periods with di-(2-ethylhexyl) phthalate or with hypolipidaemic agents. Toxicol Lett.

[b28-ehp0115-001029] Qatanani M, Zhang J, Moore DD (2005). Role of the constitutive androstane receptor in xenobiotic-induced thyroid hormone metabolism. Endocrinology.

[b29-ehp0115-001029] Rozati R, Reddy PP, Reddanna P, Mujtaba R (2002). Role of environmental estrogens in the deterioration of male factor fertility. Fertil Steril.

[b30-ehp0115-001029] Shimada N, Yamauchi K (2004). Characteristics of 3,5,3′-triiodo-thyronine (T_3_)-uptake system of tadpole red blood cells: effect of endocrine-disrupting chemicals on cellular T3 response. J Endocrinol.

[b31-ehp0115-001029] Silva MJ, Barr DB, Reidy JA, Malek NA, Hodge CC, Caudill SP (2004a). Urinary levels of seven phthalate metabolites in the U.S. population from the National Health and Nutrition Examination Survey (NHANES) 1999–2000. Environ Health Perspect.

[b32-ehp0115-001029] Silva MJ, Malek NA, Hodge CC, Reidy JA, Kato K, Barr DB (2003). Improved quantitative detection of 11 urinary phthalate metabolites in humans using liquid chromatography-atmospheric pressure chemical ionization tandem mass spectrometry. J Chromatogr B Analyt Technol Biomed Life Sci.

[b33-ehp0115-001029] Silva MJ, Reidy JA, Preau JL, Samandar E, Needham LL, Calafat AM (2006a). Measurement of eight urinary metabolites of di(2-ethylhexyl) phthalate as biomarkers for human exposure assessment. Biomarkers.

[b34-ehp0115-001029] Silva MJ, Samandar E, Preau JL, Needham LL, Calafat AM (2006b). Urinary oxidative metabolites of di(2-ethylhexyl) phthalate in humans. Toxicology.

[b35-ehp0115-001029] Silva MJ, Slakman AR, Reidy JA, Preau JL, Herbert AR, Samandar E (2004b). Analysis of human urine for fifteen phthalate metabolites using automated solid-phase extraction. J Chromatogr B Analyt Technol Biomed Life Sci.

[b36-ehp0115-001029] Surks MI, Ortiz E, Daniels GH, Sawin CT, Col NF, Cobin RH (2004). Subclinical thyroid disease: scientific review and guidelines for diagnosis and management. JAMA.

[b37-ehp0115-001029] Teass AW, Biagini RE, DeBord G, Hull RD, Eller PM (1998). Application of biological monitoring methods. NIOSH Manual of Analytical Methods.

[b38-ehp0115-001029] U.S. EPA (1997). Special Report on Environmental Endocrine Disruption: An Effects Assessment and Analysis.

[b39-ehp0115-001029] Vander A, Sherman J, Luciano D (1998). Human Physiology: The Mechanisms of Body Function.

[b40-ehp0115-001029] Welshons WV, Thayer KA, Judy BM, Taylor JA, Curran EM, vom Saal FS (2003). Large effects from small exposures. I. Mechanisms for endocrine-disrupting chemicals with estrogenic activity. Environ Health Perspect.

[b41-ehp0115-001029] Wenzel A, Franz C, Breous E, Loos U (2005). Modulation of iodide uptake by dialkyl phthalate plasticisers in FRTL-5 rat thyroid follicular cells. Mol Cell Endocrinol.

[b42-ehp0115-001029] Zoeller RT (2005). Environmental chemicals as thyroid hormone analogues: new studies indicate that thyroid hormone receptors are targets of industrial chemicals?. Mol Cell Endocrinol.

